# Integrated UPper limb and Language Impairment and Functional Training (UPLIFT) after stroke: study protocol for an umbrella Bayesian Optimal Phase IIa clinical trial

**DOI:** 10.1136/bmjno-2025-001212

**Published:** 2025-09-17

**Authors:** Kathryn S Hayward, Geoffrey Donnan, Erin Godecke, Anna Balabanski, Ruth Barker, Julie Bernhardt, Sandra Brauer, Amy Brodtmann, Emily Brogan, Sonia Brownsett, Paul Chapman, David Copland, Elise Cowley, Emily Dalton, Fiona Ellery, Paul Fink, Carlos Garcia Esperon, Annie J Hill, Heidi Janssen, Siobhan Kavanagh, Timothy Kleinig, Liudmyla Olenko, Joanne JE Quek, Trevor Russell, Moira Smith, Lillian Taylor, Vincent Thijs, Claire Tucak, John Turner, Declan Wode, Andrew Wong, Bronwyn Williams, Bruce C V Campbell, Leonid Churilov

**Affiliations:** 1 Departments of Physiotherapy and Medicine, The University of Melbourne, Melbourne, Victoria, Australia; 2 Department of Medicine, The University of Melbourne, Melbourne, Victoria, Australia; 3 Melbourne Brain Centre and Department of Neurology, The Royal Melbourne Hospital, Melbourne, Victoria, Australia; 4 Perron Institute for Neurological and Translational Science, Nedlands, Western Australia, Australia; 5 Sir Charles Gairdner Osborne Park Health Care Group, Nedlands, Western Australia, Australia; 6 Edith Cowan University, Joondalup, Western Australia, Australia; 7 Melbourne Medical School and Department of Neurology, The Royal Melbourne Hospital, Melbourne, Victoria, Australia; 8 Monash University School of Translational Medicine, Melbourne, Victoria, Australia; 9 Cairns Institute, James Cook University, Smithfield, Queensland, Australia; 10 Stroke Theme, The Florey Institute of Neuroscience and Mental Health, Heidelberg, Victoria, Australia; 11 School of Health and Rehabilitation Sciences, The University of Queensland, Brisbane, Queensland, Australia; 12 Department of Neurology, The Royal Melbourne Hospital, Melbourne, Victoria, Australia; 13 The Florey Institute of Neuroscience and Mental Health, Parkville, Victoria, Australia; 14 Department of Speech Pathology, Fiona Stanley Hospital, Murdoch, Western Australia, Australia; 15 School of Health and Rehabilitation Sciences and Queensland Aphasia Research Centre, The University of Queensland, Brisbane, Queensland, Australia; 16 Rehabilitation Unit, Townsville University Hospital, Townsville, Queensland, Australia; 17 STARS Education and Research Alliance, Metro North Hospital and Health Service, Herston, Queensland, Australia; 18 Department of Physiotherapy, The University of Melbourne, Melbourne, Victoria, Australia; 19 Department of Occupational Therapy, The Royal Melbourne Hospital, Melbourne, Victoria, Australia; 20 Lived experience research partner, Melbourne, Victoria, Australia; 21 Department of Neurology, John Hunter Hospital, Newcastle, New South Wales, Australia; 22 The University of Newcastle Australia, Newcastle, New South Wales, Australia; 23 Hunter Medical Research Institute, Newcastle, New South Wales, Australia; 24 Aphasia CRE and Department of Speech Pathology, La Trobe University, Melbourne, Victoria, Australia; 25 School of Medicine and Public Health, and School of Health Sciences, The University of Newcastle, Newcastle, New South Wales, Australia; 26 Heart and Stroke Program, Hunter Medical Research Institute, Newcastle, New South Wales, Australia; 27 Department of Neurology, Royal Adelaide Hospital, Adelaide, South Australia, Australia; 28 Department of Medicine, The University of Adelaide, Adelaide, South Australia, Australia; 29 RECOVER Injury Research Centre, The University of Queensland, Brisbane, Queensland, Australia; 30 College of Healthcare Sciences, James Cook University, Townsville, Queensland, Australia; 31 La Trobe University, Melbourne, Victoria, Australia; 32 Melbourne Medical School-Austin Campus, The University of Melbourne, Melbourne, Victoria, Australia; 33 Medical School, The University of Western Australia, Perth, Western Australia, Australia; 34 Lived experience research partner, Townsville, Victoria, Australia; 35 Alliance Rehabilitation, Townsville, Queensland, Australia; 36 Department of Neurology, Royal Brisbane and Women’s Hospital, Brisbane, Queensland, Australia

**Keywords:** APHASIA, REHABILITATION, STROKE, MOTOR CONTROL

## Abstract

**Introduction:**

One in six stroke survivors continue to experience arm and language disability at 3 months post-stroke. This study aims to identify which model(s) of integrated UPper limb and Language Impairment and Functional Training (UPLIFT) show promise for people 3 months to 24 months post-stroke. We hypothesise that at least one promising UPLIFT model of rehabilitation will be identified.

**Methods and analysis:**

This is an adaptive Phase IIa master protocol umbrella design that includes four simultaneous Bayesian Optimal Phase II studies to evaluate individual UPLIFT interventions against prespecified objective performance criteria. The intervention is upper limb and language training at 2 or 4 hours/day, 5 days/week for 4 weeks, delivered either in person (severe stratum) or via telerehabilitation (mild–moderate stratum). Up to 160 adult participants will be recruited across six metropolitan/regional university or healthcare hubs spanning five Australian states. Baseline and post-intervention assessments are blinded. A promising response is defined as a composite binary outcome combining indicators of promise of efficacy, safety and feasibility. For each UPLIFT intervention, the proportion of participants with a promising response will be monitored at three equally spaced, predefined interim stopping points and one final analysis point (n=40 participants/study). An intervention will be stopped if too few promising responses are observed.

**Ethics and dissemination:**

Ethical approval was obtained from The Royal Melbourne Human Research Ethics Committee. All participating sites obtained local governance approval. All recruited participants will provide informed consent. Trial results will be disseminated through peer-reviewed publications and presented at major stroke and rehabilitation conferences.

**Trial registration number:**

ACTRN12622000373774.

**WHAT IS ALREADY KNOWN ON THIS TOPIC**

- High-dose upper limb or language rehabilitation can promote recovery after stroke—even in the late subacute (>3 months) and chronic (>6 months) phases of recovery.

**WHAT THIS STUDY ADDS**

- This trial will identify if any integrated UPLIFT (UPper limb and Language Impairment and Functional Training) intervention dose(s) have sufficient promise to take forward to a seamless Phase IIb–III trial.

**HOW THIS STUDY MIGHT AFFECT RESEARCH, PRACTICE OR POLICY**

- This trial implements a novel early phase master protocol adaptive umbrella trial process to screen potential interventions and advance the generation of evidence in stroke recovery to avoid progression of futile treatments to late phase testing. This process is translatable to test pharmacological and non-pharmacological interventions for neurological conditions.

## Introduction

 One in six (~15%) survivors need assistance to complete arm (eg, grasping) and language (eg, conversing) functions 3 months post-stroke.^1^ Enabling long-term arm and language recovery are priorities for better living after stroke.^2^ Failure to address these limitations during community living results in unmet needs.^2^ The potential for recovery of arm and language functions can persist into the late-subacute (>3 months) and chronic (>6 months) phases of recovery post-stroke^3^,^5^ often via community rehabilitation programmes.^6^ The magnitude of gain across both upper limb^5 7 8^ and language^4^ functions appears to reduce as time post-stroke increases. Greatest gains have been observed over weeks to months rather than years post-stroke. It is critically important to reduce potential heterogeneity introduced by post-stroke disability chronicity in the context of a clinical trial. Focusing on the first 24 months post-stroke and defining subpopulations may allow the field to attempt to challenge the recovery window boundary while minimising potential heterogeneity when testing new intervention approaches.

Treatment targeting one functional limitation may improve another. Preliminary evidence has demonstrated that upper limb intervention leads to improvements in both upper limb and language outcomes^9 10^; motor cortex stimulation can effect language (aphasia) outcomes^11^; and that sequential upper limb and language sessions may enhance recovery of both.^12^ An integrated intervention approach that directly harnesses interrelated arm and language neural processes could be key to unlocking better recovery post-stroke. Integrated function reflects our real-world. For example, writing places demands on the motor (hold the pen) and language (produce the words) systems. Despite this, current care largely treats each functional limitation in isolation.

This trial of integrated intervention seeks to enhance recovery by considering dose, subpopulation and mode of integrated intervention. The current dose of upper limb or language therapy in the Australian outpatient, community living population is poorly reported. Pragmatically, it can range from no therapy through to daily therapy depending on post-stroke needs, availability of services and access to services. In clinical trials, the largest upper limb improvements in activity have been demonstrated for interventions providing greater than 2 hours/day over 4–5 days/week.^13^ The largest language improvements have been linked to 2–4 hours of intervention given over 4–5 days/week.^4^ Therefore, a minimum of 2 hours is an essential starting dose for trials aiming to improve either upper limb or language recovery.^13^ Stroke is heterogeneous and subpopulations are an important consideration. The ability to activate a muscle (eg, flicker of movement) as compared with picking up a cup indicates a different level of functional ability, as does the ability to say a single word compared with a simple sentence. Integration of subpopulation stratum based on function can help to move the field from one-size-fits-all towards tailored and targeted treatment programmes. Finally, the mode of delivery (eg, in-person, telerehabilitation) can be modified based on functional level. In the context of severe functional limitations (eg, flicker of movement or single word utterance), it may be more challenging to deliver complex and high dose interventions via telerehabilitation and in-person may be warranted. Enriching our understanding of who benefits from what intervention mode could enhance how post-stroke rehabilitation resources are used and how people can access services (eg, rural and remote areas).

With limited understanding of the optimal dose and safety profile of an integrated intervention, immediately initiating a late phase trial may expose many patients to a subtherapeutic treatment or potentially lead to harm.^1^ A Phase IIa trial is therefore most appropriate to generate data on dose, safety and feasibility. Recent trial design innovations, including a single overarching trial structure (ie, master protocol) within an umbrella design,^14^ provide an opportunity to test a given intervention (dose, mode) within a subpopulation (severity) to identify the most promising intervention(s) to take forward to a later phase trial. Each treatment:subpopulation (ie, study) can be driven by participant response at predefined interim stopping points^14^ that should exceed a prespecified objective performance criterion, that is, a value that is based on previously available relevant data or meta-analysis.^15^ Therefore, in such a design, comparison is not between groups but rather to the specified value, so that none, some or all interventions tested can demonstrate sufficient promise to progress to the next phase of testing. A good participant response can integrate dose, safety and signal of efficacy consistent with the purpose of a Phase IIa trial. If too few promising participant responses are observed, the intervention can be stopped, and future participants directed into more promising interventions. This can directly benefit current trial participants and attempt to fast-track the discovery process compared with conducting multiple separate trials.^14^

We will undertake a Phase IIa umbrella trial of integrated UPper limb and Language Impairment and Functional Training (UPLIFT) during community living after stroke. The aim of this umbrella, multicentre clinical trial is to identify the most promising integrated UPLIFT model for people 3 months to 24 months post-stroke. An umbrella design provides a single overarching master protocol structure to simultaneously evaluate individual UPLIFT interventions (studies) to identify the most promising approach. We hypothesise that at least one promising UPLIFT model of rehabilitation will be identified that can be taken forward to a Phase IIb–III trial.

## Methods and analysis

### Design

**Figure 1:**
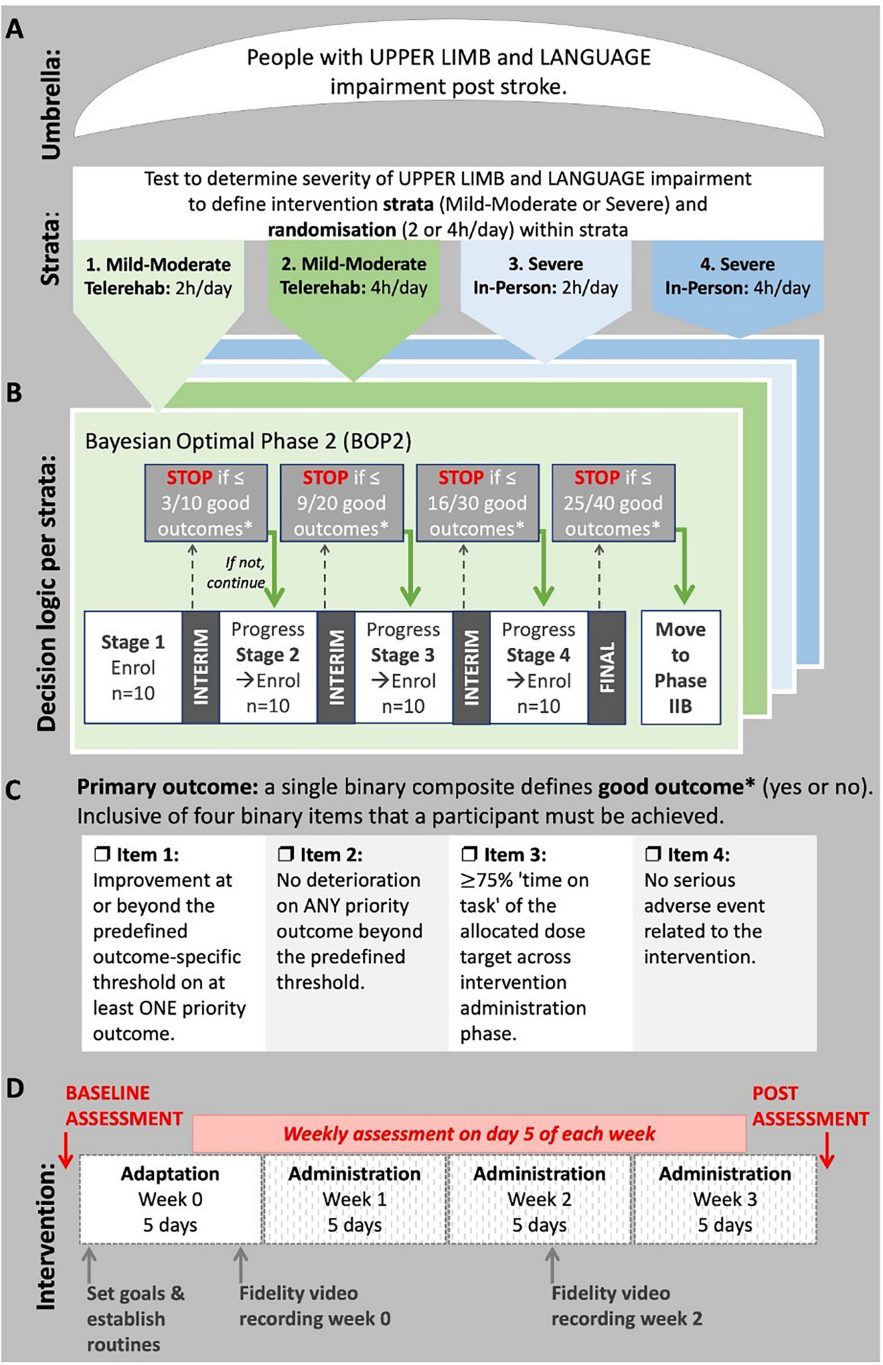
UPLIFT (UPper limb and Language Impairment and Functional Training) trial overview. (**A**) Umbrella design, strata and randomisation groups. (**B**) BOP2 decision logic for each individual stratum. (**C**) Primary outcome definition. (**D**) Intervention overview including assessment and fidelity monitoring.

This is a multicentre, assessor-blinded, adaptive umbrella Bayesian Optimal Phase IIa (BOP2) trial reported per SPIRIT (Standard Protocol Items: Recommendations for Interventional Trials)^16^ and adaptive trial extension guidelines.^17^ This trial includes four simultaneous studies to evaluate individual UPLIFT interventions against prespecified objective performance criteria.^15^ Figure 1 outlines trial design, decision logic and intervention for the four studies.

### Participant population

Up to 160 people with stroke will be recruited across six metropolitan and regional hubs across five Australian states. Eligibility criteria are defined in box 1.

Box 1 **Eligibility criteria**

**Inclusion**

- Adults aged 18 years or above.
- Clinical diagnosis of hemispheric stroke (ischaemic/haemorrhagic) between 3 months and 24 months prior to recruitment.
- If repeat stroke, no residual arm or language deficit from prior stroke(s).
- Presence of stroke-related upper limb impairment (minimum flicker of movement) and language (aphasia) impairment (minimum single interpretable word output).
- Able to follow single-stage commands.
- Able to provide informed consent, or make medical decisions, if required.
- Appropriate candidate for home therapy if required, and willingness to use required technology.

**Exclusion**

- Baseline score(s) that prevent required change on at least one priority outcome.
- No residual upper limb or language stroke-related deficit.
- Severe comorbid medical illness, eg, active intervention for cancer; concomitant neurological condition, eg, Parkinson’s disease; psychiatric illness, eg, uncontrolled bipolar disorder; other diagnosis deemed by the investigator to make a participant unlikely to be able to fully participate in all study procedures, eg, pain on movement.
- Life expectancy <3 months.

### Participant identification and consent

Potential participants can self-refer via the Stroke Foundation of Australia EnableMe consumer platform (https://enableme.org.au/community/research) or a clinician could refer with stroke survivor consent. A brief aphasia-friendly study flyer is available in written and aphasia-friendly video (https://www.youtube.com/watch?v=piuyMFZsepg) formats. All referrals are reviewed by the University of Melbourne UPLIFT Trial Central Team who conduct an email or phone prescreen followed by a video consultation to confirm eligibility (ie, upper limb and language function visual examination, check telehealth capacity if required). All potential participants are provided information in written (standard and aphasia-friendly) and video (standard https://youtu.be/9sLgo4nIakY and aphasia-friendly https://youtu.be/L9OaWALB2t0) formats. Ethical approval was granted to assess priority outcomes (defined below) during screening to ensure that the required change could be achieved per eligibility criteria. All participants or their representative provide written informed consent. Representative consent is not available at New South Wales or Western Australian sites.

### Primary outcome

A single binary composite primary outcome (achieved good outcome: yes or no) will be applied to define a good outcome. A good outcome is comprised of four binary items that are achieved at an individual participant level:

1. Improvement at or beyond the predefined outcome-specific threshold on at least ONE priority outcome, AND
2. No deterioration on ANY priority outcome beyond the predefined threshold, AND
3. Greater than or equal to 75% time-on-task of the allocated dose target across the intervention administration phase, AND
4. No serious adverse event related to the intervention.

The UPLIFT trial executive identified four priority outcomes based on psychometric properties and international consensus statement recommendations including the Stroke Recovery and Rehabilitation Roundtables^18^ and Research Outcome Measurement in Aphasia.^19^ Published literature informed group discussion and final selection of a corresponding predefined priority outcome-specific threshold:

- Fugl Meyer Upper Limb (impairment): change greater than or equal to 5.25 points.^20^
- Box and Block Test (activity): change greater than or equal to 5.5 blocks.^21^
- Western Aphasia Battery-Revised Aphasia Quotient (impairment): change greater than or equal to 5 points.^22^
- Communicative Effectiveness Index (CETI, activity): change greater than or equal to 10.37 points.^23^

All priority measures are performed by the participant except for the CETI. The CETI is completed by a caregiver, family member or partner of a person with aphasia to assess their functional communication abilities in everyday situations. Post-intervention CETI scoring is independent of baseline score knowledge. If a participant does not have an appropriate person to complete the CETI, the remaining three priority outcomes will only be used to inform good outcome achievement.

### Secondary outcomes

Secondary outcomes are collected at baseline and post-intervention (table 1). A formal process evaluation is not required in this early phase trial. We included a simple evaluation to probe intervention dose, content and access barriers and enablers. All assessors are trained (review of written and audiovisual materials, virtual training session, competency scoring where available) in primary and secondary outcomes before completing any trial-related assessments.

**Table 1:** UPLIFT trial schedule of enrolment, intervention and assessments

	Study period				
	Enrolment	Baseline		Intervention	Post-intervention
TIME POINT	<Week 0	Week 0		Week 1–4	>Week 4
ENROLMENT:					
Eligibility screen	X				
Informed consent	X				
Allocation			X		
INTERVENTION:					
Mild–moderate telerehabilitation, 2 hours/day				X	
Mild–moderate telerehabilitation, 4 hours/day				X	
Severe in-person, 2 hours/day				X	
Severe in-person, 4 hours/day†*				X	
Multidimensional dose recording*				X	
Fidelity video recording				X	
Upper limb weekly assessment: Rating of Everyday Arm and hand use in the Community and Home				X	
Language weekly assessment: Visual Analogue Scale for communication				X	
ASSESSMENT†:					
Demographic Information		X			
Stroke Information		X			
modified Rankin Scale		X			X
Fugl Meyer Upper Limb Assessment*		X			X
Box and Block Test*		X			X
Western Aphasia Battery-Revised*		X			X
Communicative Effectiveness Index scale*		X			X
9-Hole Peg Test		X			X
Action Research Arm Test		X			X
Rating of Everyday Arm and hand use in the Community and Home		X			X
Discourse (video-recorded)		X			X
Reaching for a cup (video-recorded)		X			X
Trail Making Test B		X			X
Clock Drawing Task		X			X
Boston Naming Test		X			X
Stroke and Aphasia Quality of Life Scale-39		X			X
National Institute of Health Stroke Scale		X			
Process evaluation		X			X
Adverse events*		X		X	X

* Contributes to single binary composite primary outcome.

† Baseline assessments by blinded assessor completed 5 days (±2 days) prior to Intervention onset. Post assessments by blinded assessor completed within 5 days (±2 days) of Week 4 Day 5.

### Safety outcomes

All adverse events will be recorded from the time of consent until the post assessment has been completed (end of study). Adverse event documentation will include start and stop dates, action taken, outcome, intensity and relationship to study intervention (causality). Adverse events will be classified as serious or not and intervention related or not. In addition, all serious adverse events will include a rating of severity, causality and expectedness. All safety events (serious adverse events and adverse events) will be reviewed and a final classification documented by the medical monitor.

### Randomisation and blinding

Randomisation to 2 or 4 hours of intervention dose is within strata (severe, mild–moderate) to achieve better representativeness of individual studies as well as better sequential balance within individual studies given the frequent nature of interim analyses in this early phase design. Randomisation is not used to enable statistical inference of comparative effect as the nature of this design does not assume comparison of various intervention doses. Randomisation is via a centralised allocation system designed by the trial statistician (LC). Participants allocated to the severe strata must meet the minimum entry criteria (box 1). Participants allocated to the mild–moderate strata must: (1) have baseline scores that permit the required threshold change on all priority outcomes, (2) be able to pick up an empty standardised cup and raise it off the table for 5 s without drift of the cup, hand, wrist or elbow to the table and (3) be able to say a correct, basic sentence, for example, ‘I drink water’. Upper limb and language tasks for strata severity rating are video-recorded and evaluated by two study personnel. Initial raters discuss the outcome if there is a discrepancy before a third person reviews the video recording. Each BOP2 study can have up to four cohorts (n=10) and no cohort can have more than 50% of participants who are more than 12 months post-stroke. On completion of strata severity rating, participants are randomly allocated within each stratum to 2 or 4 hours/day. Forced randomisation is possible in the event a clinical site faced a situation when an eligible trial participant was to be randomised to a treatment not available to be delivered per protocol at the site.^24^

### Sample size calculation

While Bayesian in nature, our design ensures appropriate frequentist characteristics (power=0.8, alpha=0.05). The sample size was estimated using an online BOP2 design tool.^25^ Allowing for three equally spaced interim stopping points (n=10, n=20, n=30) and a final decision point analysis (n=40), a sample size of 40 participants per study was estimated using the null hypothesis rate of upper limb and language recovery of 0.5 vs alternative hypothesis rate of recovery of 0.7.^26^

### Intervention

The UPLIFT intervention description is per the TIDieR (template for intervention description and replication) statement.^27^ All intervention is consistent with usual care as recommended in the Australian Living Clinical Practice Guidelines.^28^ There are three session foci: (1) upper limb motor impairment (eg, target strength, control, coordination and flexibility) and functional task (eg, stabilisation, reach, grasp, manipulation and release) training to maximise transition to real-world use; (2) language impairment (ie, production at the word, sentence and conversational level in comprehension and speech) and communication in context (functional communicative effectiveness in relevant everyday situations through multiple modalities such as gestures, speech and writing) training; and (3) integrated upper limb and language impairment and functional training to maximise the potential for therapeutic synergy (synchronously/asynchronously). Session content is individually tailored to each participant based on their goals (4 weeks, 3 months and dream, ie, no time limit) and daily routines (upper limb and language use during a typical day) that are established in Week 0 Session 1. Content is adapted within and between sessions to ensure optimal challenge based on a participant’s daily performance and to maximise and maintain engagement. In the first session, the participant and treating therapist discuss the participants usual upper limb and language daily routines (from waking up through to going to bed, probing how the upper limb or language is used in all activities) and establish goals (4 weeks/3 months/dream). Materials are consistent with a standard outpatient rehabilitation facility and include access to training equipment, electrical stimulation units, height adjustable bed and table. We do not use any robotic devices. Participants in the severe strata receive intervention in person at a university or health clinic, while the mild-moderate strata receive all intervention via telerehabilitation (Zoom) in a setting with appropriate internet and safety set-up, for example, quiet space in their home. Over 4 weeks, a minimum of 20 (of total 80) sessions each of upper limb, language and integrated treatment are provided, leaving 20 flexible sessions, for example, to participant’s goals or staffing availability. Dose within each session is reported in real-time using a custom-built repeated instrument REDCap form consistent with a multidimensional dose articulation framework.^29^ Table 2 describes how all dose dimensions are applied and measured within the UPLIFT protocol. Participants can do up to 2 hours/week of upper limb and/or language therapy intervention outside UPLIFT. A small amount of concomitant therapy is allowed to ensure participants do not lose access to services because of trial participation. All UPLIFT intervention is provided by physiotherapists, occupational therapists, speech pathologists or allied health assistants who are trained in study procedures (ie, study protocol, dose recording, adverse event reporting) via review of written and audiovisual materials, and attendance at a virtual training session.

**Table 2:** Multidimensional dose articulation framework dimensions

Item	Dose dimension	UPLIFT protocol
1	Duration	Completion within a minimum of 4 weeks and maximum of 5 weeks.
2	Days	Monday to Friday; minimum 20 weekdays, maximum 25 weekdays to allow for, eg, public holidays, medical appointments, staff illness.
3	Sessions	Four sessions/day. Focus on upper limb, language or integrated content.
4	Session length	30 min if 2 hours/day or 60 min if 4 hours/day.
5	Session density	Ratio of time-on-task to time-off-task.
6	Episode(s) length	Equates to a task completed and how long for, ie, time-on-task Time on task **includes**: Successful/unsuccessful repetitions Appropriate rest to recover between repetitions or sets Task demonstration Task-related instructions Task-related feedback Weekly assessments Time on task **does not include**: General conversation (unless language task) Set-up of environment where participant is not engaged Transfers not related to the task Scheduling of appointments Discussing previous sessions or post-session effects Toilet breaks Scheduled rest or time off task >2 mins Adverse event discussions Telehealth technical issues, eg, connection issues
7	Episode(s) difficulty	Consistent ratings used for all episodes of all sessions. **Level A**: Consistent reliance on supports to complete task. **Level B**: Require support to complete the task more often than not. **Level C**: Mostly independent in task completion. **Level D**: Mostly independent in task completion with challenges added. Support examples: gravity-eliminated, electrical stimulation. Challenge examples: load, gravity, speed.
8	Episode(s) intensity	Successful repetitions, unsuccessful repetitions, summed total repetitions. Task goal, eg, wrist extension to target. Successful repetitions: any attempt that meets the task, eg, wrist extension to reach the target. Unsuccessful repetitions: any repetition less than the task, eg, wrist extension that did not reach the target. Total repetitions: summed successful and unsuccessful repetitions.

UPLIFT, UPper limb and Language Impairment and Functional Training.

### Fidelity

All data are entered into a custom-built electronic case record form in REDCap and checked remotely (goal within two working days) by UPLIFT central staff. Site-specific staff address all data queries. A file note with reason(s) is included in REDCap whenever a session is >10% shorter than the target dose (ie, <27 min for 30 min session or<54 min for 60 min session) or if technical issues arise during telerehabilitation. The entirety of two intervention days (10% of duration) are video-recorded (Zoom): Week 0 Day 5 (prior to Week 1 data collection commencement) and Week 2 Day 3 (mid-intervention).

### Statistical analysis

A statistical analysis plan will be completed prior to database lock. A participant will be deemed to have not achieved a good outcome if withdrawal is for trial-related reasons (eg, intervention load). A participant will be replaced if withdrawal is for non-trial related reasons (eg, COVID-19). An independent (LO) member will prepare all data summaries for interim and final analyses. Two people will review interim data (KSH content expert and LC statistical expert), protocol deviations and file notes against a priori defined rules to rate each binary item of the composite primary outcome as achieved or not. For efficacy, the change between baseline and post-intervention is calculated for each priority outcome to determine if there is improvement or decline at or beyond the predefined outcome-specific threshold. The first binary item is considered achieved by a participant if at least one priority outcome improves beyond the predefined threshold. The second binary item is considered achieved if the participant does not decline beyond the threshold for any priority outcome. For feasibility, the proportion of time-on-task is averaged across the intervention administration period (Weeks 1/2/3). The third binary item is considered achieved by a participant if their average time-on-task is ≥75%. For safety, all serious adverse event outcomes are reviewed for intervention relatedness. The fourth binary item is considered achieved by the participant if they have no intervention-related serious adverse event. If all four binary items are rated achieved, then a participant is deemed to have made a good outcome. Recruitment to a given study will continue (up to a maximum of n=40) if sufficient participants achieve a good outcome on the single binary composite primary outcome at interim (see figure 1). A study will be stopped if insufficient participants achieve a good outcome at interim. Online supplemental A provides an interim analysis template. All secondary outcomes will be reported at baseline, post-intervention and baseline-post-intervention change (median, SD).

### Trial governance and funding

The UPLIFT trial sponsor is the University of Melbourne Australia. The UPLIFT trial is governed by an executive committee (Chairs KSH/GD; all study members to monitor overall trial operations), steering committee (Chair KSH; all site principal investigators to monitor site operations) and a central coordinating team (Chairs KSH/JQ, oversee the day-to-day trial operations). An independent Data Safety Monitoring Committee (DSMC) adheres to a predefined charter and includes three experienced multidisciplinary stroke clinicians and trialists (neurologist, geriatrician, physiotherapist). The trial statistician (LC) and lead investigator (KSH) provide a trial report at each DSMC meeting (including all safety outcomes and their rating by the medical monitor), which are triggered when an individual BOP2 study has recruited n=20 or at minimum annually. Steering committee, DSMC and central coordinating team all report to the executive committee. This trial is funded by the Medical Research Future Fund (MRF2007425).

### Patient and public involvement

The UPLIFT trial governance structure includes a lived experience group (Chairs EG/RB, three people with stroke, one carer) who contribute to the development and review of trial materials. This includes trial information that were established in written and video formats. This committee also co-designed a participant-level study outcome summary.

## Ethics and dissemination

Ethical approval was obtained from The Royal Melbourne Human Research Ethics Committee (HREC/79639/MH-2021). All participating sites obtained local governance approval. The trial was prospectively registered with the Australian and New Zealand Clinical Trial Registry (ACTRN12622000373774 on 3 March 2022) and endorsed by the Australasian Stroke Trials Network. First patient, first visit occurred on 24 May 2022. Last patient, last visit anticipated to occur in mid-2026. Trial results will be disseminated through peer-reviewed publications, and major stroke and rehabilitation conference presentations. Study consent forms include capacity to opt-in to non-identifiable study data being shared with other researchers working on closely related future projects that have received the relevant ethical approvals. Only participants who have opted in to data sharing will be eligible for individual participant data sharing. All data access requests can be made to the UPLIFT trial executive (uplift-trial@unimelb.edu.au).

## Discussion

This trial includes innovations in trial design, intervention approach, trial infrastructure and materials that may advance how stroke recovery trials are conducted.

We employed an umbrella master protocol that serves to promote a more efficient trial process than previously undertaken, while adhering to a discovery pipeline approach.^30^ Efficiency is demonstrated by interim analyses that can result in stopping any UPLIFT intervention study that does not demonstrate sufficient promise and only continuing UPLIFT intervention studies that do demonstrate promise. For the field of stroke recovery, this approach can enable more rapid screening of potential interventions. Stopping should only be interpreted as evidence that an intervention does not show promise in the study sample recruited (ie, severity, time post-stroke) under the conditions tested (ie, duration, session length, mode). Continuing to recruit under promising conditions will generate sufficient data to generate a sample size estimate for a subsequent Phase IIb–III trial. Together, this approach will efficiently and rigorously evaluate the proposed UPLIFT intervention and guide the identification of evidence-based stroke recovery treatments.

The UPLIFT trial uniquely integrates treatment for two systems commonly impacted by stroke within a high-dose paradigm. The principles underpinning our intervention are not dissimilar to enriched rehabilitation (environmental enrichment paired with high dose motor training) that is efficacious in preclinical models of stroke.^31^,^35^ Environmental enrichment targets multiple systems simultaneously (physical, cognitive and social) as does UPLIFT (physical and language), and both approaches deliver high dose training. UPLIFT builds on the concept that a multisystem approach to treatment delivered at high doses may accelerate recovery.

The UPLIFT trial group has established infrastructure that enables dose to be recorded real-time. This infrastructure is critical for several reasons. First, it supports efficient and detailed documentation to understand what dose was provided to each participant. Second, it enables fidelity monitoring to be completed daily; not possible using traditional paper-based records stored locally. Third, it reduces the environmental impact and administrator load of documenting high-dose (2 or 4 hours/day) protocols. Finally, real-time recording can support multisite national and international stroke recovery trials, which are currently infrequent.^36^,^38^

This trial does have limitations. It is Phase IIa and cannot provide definitive evidence. Recruitment is constrained to a predefined period post-stroke (3-months to 2-years post-stroke) using broad but specific functional boundaries. However, each study is designed to produce evidence of signal of efficacy, should it exist, for any UPLIFT intervention dose that can be used to inform the design of an appropriate Phase IIb–III trial.

## Supplementary material

10.1136/bmjno-2025-001212 online supplemental file 1
