# Supplementary material for: Integrated UPper limb and Language Impairment and Functional Training (UPLIFT) after stroke: study protocol for an umbrella Bayesian Optimal Phase IIa clinical trial
Source: BMJ Neurol Open. 2025 Sep 17;7(2):e001212. doi: 10.1136/bmjno-2025-001212 (PMC12458844; doi:10.1136/bmjno-2025-001212)
Supplement: online supplemental file 1 [file bmjno-7-2-s001.pdf]

Supplemental materials for: Integrated UPper limb and Language Impairment and Functional Training (UPLIFT) after stroke: Study protocol for an umbrella Bayesian Optimal Phase IIa clinical trial.

Supplemental A: Template for interim review reporting

[intervention]											
Participant	Block 1 Outcome		Participant	Block 2 Outcome		Participant	Block 3 Outcome		Participant	Block 4 Outcome	
1 (Part. #)	A	B	1 (Part. #)	A	B	1 (Part. #)	A	B	1 (Part. #)	A	B
	C	D		C	D		C	D		C	D
2 (Part. #)	A	B	2	A	B	2	A	B	2	A	B
	C	D		C	D		C	D		C	D
3 (Part. #)	A	B	3	A	B	3	A	B	3	A	B
	C	D		C	D		C	D		C	D
4 (Part. #)	A	B	4	A	B	4	A	B	4	A	B
	C	D		C	D		C	D		C	D
5 (Part. #)	A	B	5	A	B	5	A	B	5	A	B
	C	D		C	D		C	D		C	D
6 (Part. #)	A	B	6	A	B	6	A	B	6	A	B
	C	D		C	D		C	D		C	D
7 (Part. #)	A	B	7	A	B	7	A	B	7	A	B
	C	D		C	D		C	D		C	D
8 (Part. #)	A	B	8	A	B	8	A	B	8	A	B
	C	D		C	D		C	D		C	D
9 (Part. #)	A	B	9	A	B	9	A	B	9	A	B
	C	D		C	D		C	D		C	D
10 (Part. #)	A	B	10	A	B	10	A	B	10	A	B
	C	D		C	D		C	D		C	D
Total:											

	Outcome met	A good clinical outcome is defined by achieving all of:<br>**A.** [Insert]<br>**B.** [Insert]<br>**C.** [Insert]<br>**D.** [Insert]
	Outcome not met	
Final outcome for interim:		
